# Artificial intelligence and genomic data privacy: Balancing innovation with security

**DOI:** 10.22099/mbrc.2025.54335.2217

**Published:** 2026

**Authors:** Leila Kohan

**Affiliations:** Department of Biology, Ars.C., Islamic Azad University, Arsanjan, Iran

Artificial intelligence (AI) has emerged as one of the most transformative technologies in contemporary medicine and biology. Its remarkable ability to process and analyze vast amounts of biological information, particularly genomic data, has opened new avenues for personalized medicine, early disease detection, and drug discovery. Numerous studies have demonstrated that AI-based algorithms can significantly improve diagnostic accuracy, tailor therapies to individual genetic profiles, and accelerate the development of novel therapeutics.

Despite these unprecedented opportunities, serious concerns have surfaced regarding genomic data privacy. Unlike most other forms of medical information, genomic data possesses a unique and highly sensitive nature. It not only reveals the genetic identity of an individual but may also disclose information about their relatives and even future generations. Consequently, any misuse or leakage of genomic information can have far-reaching implications that extend well beyond the individual [1].

AI has the capacity to uncover hidden and complex patterns within genomic datasets, driving several key applications. In disease diagnosis, machine learning algorithms can detect early signs of disease with remarkable precision; for instance, deep learning models have been shown to improve cancer detection with notable accuracy. In personalized medicine, AI enables the development of patient-specific treatments by analyzing genetic data, thereby enhancing therapeutic effectiveness while minimizing adverse effects [2]. Furthermore, in the field of drug discovery and development, AI-driven models can screen potential compounds and predict their efficacy, significantly shortening the timeline for drug development and reducing associated costs [3]. These advances illustrate the extent to which the future of medicine and genomics is intertwined with AI technologies. However, the very progress that drives innovation simultaneously gives rise to pressing challenges.

As the use of AI in genomics expands, concerns about data protection are becoming increasingly urgent. Even anonymized genomic data carries the risk of re-identification, particularly when combined with other datasets such as clinical or digital health records. In addition, there is the potential for misuse: genomic data could be exploited by corporations, insurers, or other entities for unethical or discriminatory purposes, such as in employment decisions or insurance policies. These risks are further compounded by the potential erosion of public trust. If individuals lose confidence in the safety of their genomic data, participation in biobanks and genomic research projects will decline, ultimately jeopardizing scientific progress. For these reasons, genomic data is rightly regarded as the most sensitive category of health information, requiring robust measures of protection [4, 5].

To harness the full potential of AI in genomics while safeguarding individual rights, it is essential to establish balanced strategies that promote both innovation and security. Updated legal frameworks are crucial in this regard. While regulations such as the General Data Protection Regulation (GDPR) in Europe represent important steps forward, many regions still lack comprehensive policies. International cooperation and harmonized legislation are urgently needed to ensure consistent standards for data governance across borders.

Equally important are advanced data protection technologies. Approaches such as encryption, federated learning, and differential privacy allow researchers to analyze genomic data without transferring or exposing raw information, thereby reducing the risk of breaches or unauthorized access. Education and awareness also play a vital role. Researchers, clinicians, and even patients must be informed about the ethical responsibilities associated with handling genomic data. Training in data ethics should be regarded as just as essential as training in bioinformatics or machine learning. Moreover, transparency in research is fundamental to maintaining scientific integrity and public confidence. Scientific journals and institutions should require clear and detailed reporting on how genomic data are managed, protected, and shared. Such transparency not only reinforces accountability but also helps to build and sustain trust among participants and the broader public [6, 7].

The convergence of AI and genomics represents one of the most groundbreaking scientific developments of our time, with the potential to revolutionize disease diagnosis, treatment, and prevention while advancing the vision of truly personalized medicine. However, the sustainability of this progress depends on embedding ethical and security considerations at the very core of these advancements. As members of the scientific community, we must work collectively to safeguard both innovation and human dignity. Achieving this balance will require close collaboration among researchers, policymakers, regulatory bodies, and society at large. Only through such cooperation can we fully unlock the extraordinary potential of AI in genomics without compromising privacy, trust, and the fundamental rights of individuals.
